# Influence of Intrapancreatic Fat Deposition on Regional and Total Pancreatic T1 Relaxation Times at 3.0 Tesla MRI

**DOI:** 10.3390/jimaging12050185

**Published:** 2026-04-24

**Authors:** Xiatiguli Shamaitijiang, Beau Pontre, Loren Skudder-Hill, Yutong Liu, Maxim S. Petrov

**Affiliations:** 1 School of Medicine, University of Auckland, Auckland 1023, New Zealand; 2 School of Medical Sciences, University of Auckland, Auckland 1023, New Zealand

**Keywords:** magnetic resonance imaging, T1 relaxation time, intrapancreatic fat deposition, pancreas

## Abstract

Longitudinal relaxation time (T1) can be used to assess pancreatic pathology on magnetic resonance imaging (MRI). Although pancreatic T1 values may be influenced by intra-organ fat content, regional variation within the pancreas and the impact of potential confounders have not been comprehensively examined. This study aimed to investigate the nuanced associations between intrapancreatic fat deposition (IPFD) and both regional and total pancreatic T1 relaxation times. Pancreatic T1 relaxation times were quantified with B1-corrected dual flip-angle 3D-VIBE imaging at 3.0 Tesla, whereas IPFD was measured with a high-speed, T2-corrected multi-echo sequence. Linear regression models were constructed to evaluate the association between IPFD and T1 values, with adjustment for relevant covariates. A total of 124 individuals were included in the analysis. IPFD explained 4.6% of the variance in total pancreatic T1 values, with notable regional differences: 1.0% in the head, 3.0% in the body, and 7.7% in the tail of the pancreas. In the fully adjusted model, IPFD was significantly associated with total pancreatic T1 values (*p* = 0.001), with consistent significant associations observed across all pancreatic regions: head (*p* = 0.03), body (*p* = 0.004), and tail (*p* = 0.002). These findings demonstrate that IPFD is a significant determinant of pancreatic T1 relaxation times. Accordingly, IPFD should be considered a potential confounder in pancreatic T1 assessments and accounted for when interpreting T1 relaxation times on pancreatic MRI in both research and clinical contexts.

## 1. Introduction

Excessive intrapancreatic fat deposition (IPFD) is implicated in the pathogenesis of major pancreatic diseases [1]. Moreover, the PANcreatic Diseases Originating from intrapancReatic fAt (PANDORA) hypothesis postulates that excessive IPFD is not merely an epiphenomenon but may actively contribute to various diseases arising from the pancreas, including type 2 diabetes mellitus (T2DM), chronic pancreatitis, and pancreatic cancer [2]. In parallel, magnetic resonance imaging (MRI) has enabled non-invasive quantification of both IPFD and tissue composition [3], creating an opportunity to disentangle the structural and compositional determinants of pancreatic parameters.

Among the readily available MRI parameters in clinical practice is the quantitative assessment of tissue longitudinal relaxation times (T1) using T1 mapping. This method generates pixel-by-pixel maps that reflect tissue composition—such as hydrogen atom density—enabling a more precise and objective assessment of tissue characteristics compared with conventional MRI, where tissue characterisation is based on qualitative image contrast [4,5,6,7,8]. Previous studies have reported that changes in T1 relaxation times are significantly associated with chronic pancreatitis, pancreatic cancer, and T2DM—diseases that are also characterised by excessive IPFD [5,6,7,9,10]. In addition, the reference ranges for pancreatic T1 relaxation times have been reported and shown to correlate with age [11]. However, the interpretation of pancreatic T1 remains challenging because T1 is sensitive to tissue composition, including fat content [12]. If IPFD is not accounted for, observed T1 differences between groups may reflect compositional confounding rather than disease-specific biology [13]. This issue may partly explain the inconsistency across prior pancreatic T1 studies. Previous work on the relationship between IPFD and pancreatic T1 is limited in three important ways: relatively small samples, restricted clinical cohorts, and no covariate adjustment [14]. In addition, most studies emphasised whole-pancreas or average estimates, despite the pancreas being anatomically and functionally heterogeneous [2]. Regional assessment is important because different portions of the organ vary in embryologic origin, ductal and vascular anatomy, as well as endocrine cell distribution [2]; therefore, IPFD-related T1 effects may not be spatially uniform.

Against this background, the present study aimed to investigate whether IPFD is associated with both total and regional native T1 relaxation times of the pancreas in a larger cohort representative of general population. We additionally assessed splenic T1 as a comparator tissue expected to have minimal fat-related signal confounding.

## 2. Materials and Methods

### 2.1. Study Design and Population

This cross-sectional study involved adult participants representative of the general population who underwent MRI of the pancreas for the purpose of research. Participants were excluded if they required hospitalisation within three months prior to study enrolment, had chronic pancreatitis, pancreatic cancer, or any conventional imaging abnormality of the pancreas, received steroid therapy or interventions involving the pancreas (radiological, endoscopic, or surgical), or were pregnant. Also, individuals were excluded if they had contraindications to undergoing MRI such as cognitive impairment, claustrophobia, congestive heart failure, chronic obstructive pulmonary disease severe enough to limit breath-holding, or metal implants that were not compatible with MRI [15,16]. This study was conducted according to the principles in the Declaration of Helsinki and approved by the Ethics Committee. Written informed consent was provided by all individuals.

### 2.2. Imaging Data Acquisition and Analysis

All participants underwent an abdominal MRI scan specifically for the purpose of this research, using a 3.0 Tesla MAGNETOM scanner (Siemens, Erlangen, Germany) with an 18-channel anterior coil and posterior built-in spine coil. Participants fasted before the scan and were instructed to lie supine.

**Figure 1:**
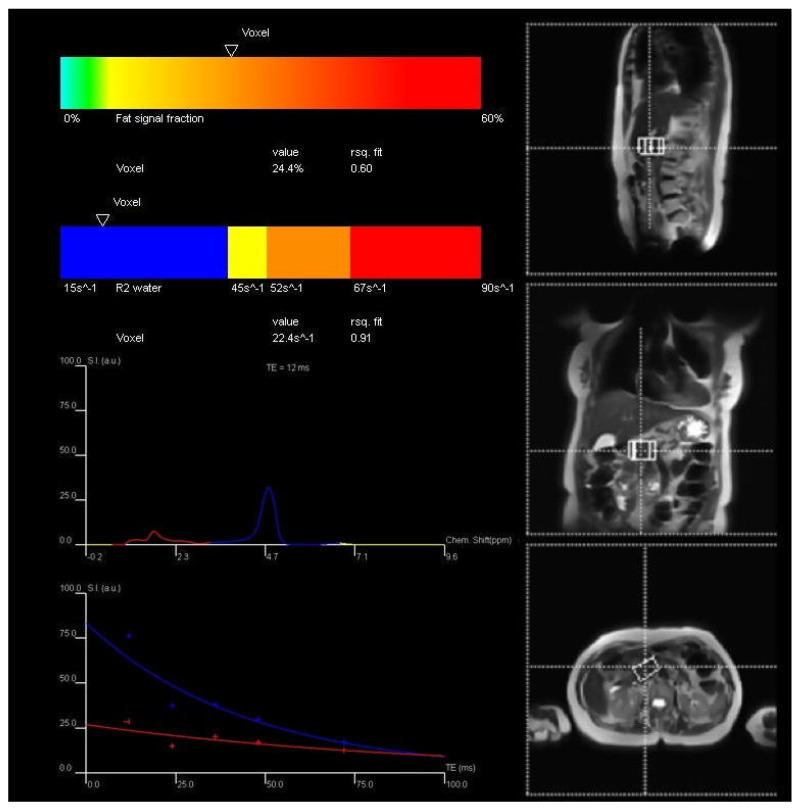
Exemplar measurement of intrapancreatic fat deposition. *Footnote*: Intrapancreatic fat deposition was determined with the use of high-speed T2-corrected multi-echo sequence. Coloured curves represent spectra and decay plots showing different fitted components. Blue indicates a slower-decaying component (water), while red corresponds to a faster-decaying component (fat). The dotted crosshair lines indicate the spatial localisation of the voxel.

The high-speed T2-corrected multi-echo (HISTO) MR spectroscopy sequence was used to measure IPFD. The acquisition voxel was placed at the border of the pancreas head and body by an experienced MR technologist. Five spectra were collected at echo times of 12 ms, 24 ms, 36 ms, 48 ms, and 72 ms in single breath-hold. The parameters were as follows: true form abdomen shim mode; voxel size = 22 × 40 × 31 mm; repetition time = 3000 ms; acquisition duration = 853 ms; flip angle = 90 degrees; and breath-hold time was 15 s [17]. The acquired spectra were processed using the inline Syngo software (Siemens Healthcare GmbH, Erlangen, Germany) to perform T2 correction and derive IPFD values (Figure 1). The resulting data were exported as DICOM files and reviewed using MicroDicom (MicroDicom, Sofia, Bulgaria).

**Figure 2:**
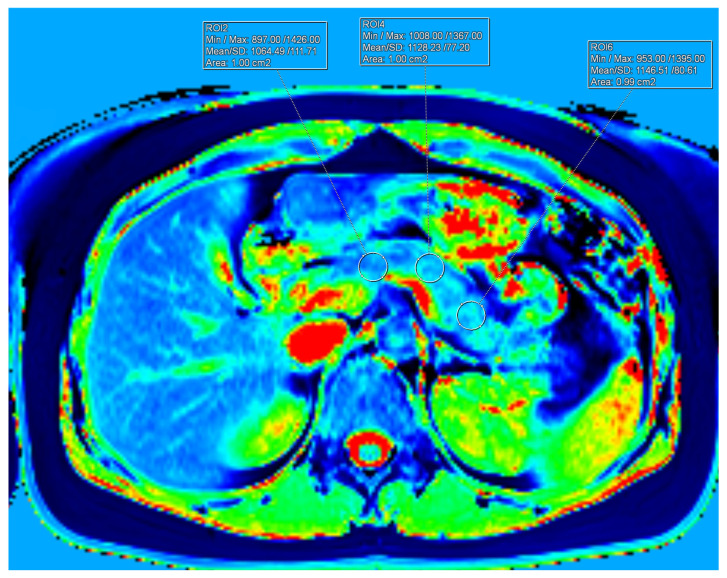
Exemplar measurements of T1 relaxation time of the pancreas. *Footnote*: The different colours in the figure represent a colour-coded T1 relaxation time map of the pancreas. Dark blue/deep blue areas indicate lower T1 relaxation times, green/yellow regions correspond to intermediate T1 values, and red/bright red areas represent higher T1 relaxation times. Three regions of interest (100 ± 2 mm^2^ each) were placed in the head, body, and tail of the pancreas.

T1 relaxation times were quantified using a 3D volumetric interpolated breath-hold examination (VIBE) sequence with the following acquisition parameters: repetition time (TR) = 3.87 ms; echo time (TE) = 1.76 ms; acceleration factor = 3; flip angles = 2° and 12°; in-plane resolution = 1.3 × 1.3 mm; and a breath-hold duration of 17 s. A total of 48 contiguous slices, each 4 mm thick, were acquired. Additionally, a low-resolution B1 map was obtained using a turbo-FLASH sequence to correct for B1 inhomogeneities. Detailed pulse sequence parameters for both T1 mapping and B1-corrected T1 mapping are described elsewhere [3]. Two slices were selected where the pancreas was centrally located along the foot–head axis to minimise partial volume effects and provide sufficient area to place three regions of interest (ROIs), each measuring 100 mm^2^, over the head, body, and tail of the pancreas. ROI placement carefully avoided inclusion of the splenic vein, arteries, inferior vena cava, duodenum, and retroperitoneal fat. The ROI for the pancreas head was positioned in line with the right-most point of the confluence of the superior mesenteric and splenic veins. The ROI for the pancreas body was aligned with the left lateral border of the lumbar vertebrae. The ROI for the pancreas tail was placed on a line perpendicular to the organ’s midline, approximately 20 mm from the distal-most point of the pancreas (Figure 2). The mean T1 value for each pancreatic region was calculated by averaging the T1 measurements from both selected slices. The overall mean T1 value of the pancreas was then derived by averaging the regional means. For the spleen, T1 was measured three times on the slice exhibiting the greatest amount of splenic parenchyma. An ROI of approximately 200 mm^2^ was placed centrally within the spleen, at least 5 mm from the organ’s edge, avoiding areas of artifact, focal lesions (if present), and major blood vessels. The mean splenic T1 was obtained by averaging these three measurements. All T1 quantification was performed using Syngo.via software (Siemens Healthcare GmbH, Erlangen, Germany).

### 2.3. Covariates

Fasting venous blood samples (>8 h) were collected on the day of the MRI scan. Glycated haemoglobin (HbA1c) was measured immediately on never frozen blood using a boronate affinity chromatography assay (Trinity Biotech, Wickow, Ireland). Fasting glucose was measured using the enzymatic colourimetric assay (F. Hoffmann-La Roche Ltd., Basel, Switzerland) [17]. High-density lipoprotein (HDL) cholesterol and triglyceride were analysed using standard assays at a tertiary referral lab. The waist–height ratio was calculated by dividing the waist by the height.

### 2.4. Statistical Analysis

All statistical analyses were performed using SPSS Statistics for Windows, version 28 (IBM, Armonk, NY, USA). Continuous variables were presented as the median and interquartile range (IQR) whereas categorical variables were reported as frequencies and percentages. Data for HbA1c, glucose, HDL cholesterol, and triglycerides were log_10_-transformed due to non-normal distribution, as assessed by the Shapiro–Wilk test.

Multivariable linear regression analyses were conducted to examine the associations between IPFD and T1 relaxation times. For each outcome (total pancreatic T1, pancreatic head T1, pancreatic body T1, pancreatic tail T1, and splenic T1), we fitted a linear model with IPFD as the primary independent variable, using the following prespecified covariate adjustments:

- Model 1: Unadjusted.
- Model 2: Adjusted for age and sex.
- Model 3: Adjusted for age, sex, ethnicity, and waist-to-height ratio.
- Model 4: Adjusted for age, sex, ethnicity, waist-to-height ratio, glucose, and HbA1c.
- Model 5: Adjusted for age, sex, ethnicity, waist-to-height ratio, glucose, HbA1c, HDL cholesterol, and triglycerides.

Collinearity was considered negligible, as all variance inflation factor values were below 5. Results were reported as β coefficients, *p* values, and 95% confidence intervals (CIs), with *p* < 0.05 considered statistically significant.

## 3. Results

### 3.1. Characteristics of Participants

A total of 124 participants aged 19 to 83 years were included in this study, comprising 57 women (46%, aged 27 to 83) and 67 men (54%, aged 19 to 71). Detailed participant characteristics are presented in Table 1.

### 3.2. Associations of Intrapancreatic Fat Deposition with T1 of the Total Pancreas and the Spleen

**Figure 3:**
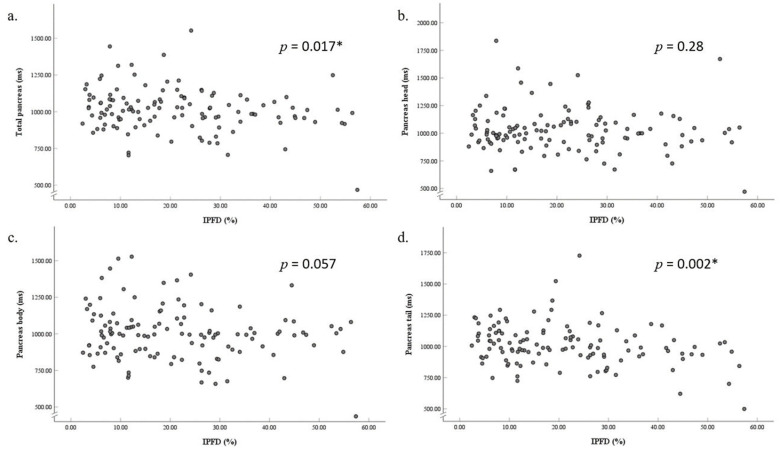
Associations between intrapancreatic fat deposition and T1 relaxation time of the total pancreas (**a**), pancreas head (**b**), pancreas body (**c**), and pancreas tail (**d**). *Footnote*: All partial residual plots were generated from unadjusted models. * Indicates statistically significant values (*p* < 0.05). *Abbreviation*: IPFD, intrapancreatic fat deposition.

**Figure 4:**
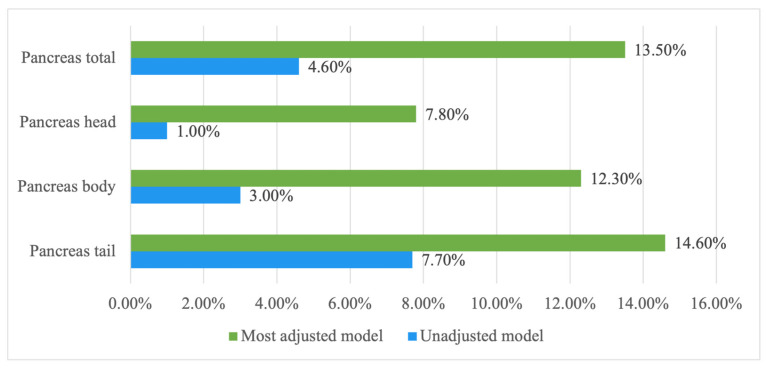
Contributions of intrapancreatic fat deposition to the variance in T1 relaxation time of the pancreas. *Footnotes*: Data are presented as percentage of intrapancreatic fat deposition that explains the variance in the T1 relaxation time of the regional and total pancreas in the unadjusted and the most adjusted models (accounting for age, sex, ethnicity, waist–height ratio, glucose, glycated haemoglobin, high-density lipoprotein cholesterol, and triglyceride).

The median T1 relaxation time of the total pancreas was 1012.75 ms (IQR: 940.58 to 1085.11 ms). A significant negative association was observed between total pancreatic T1 and IPFD in the unadjusted model (β = –2.22, *p* = 0.017) (Figure 3), as well as in all the adjusted models, including the most fully adjusted model (β = –3.71, *p* = 0.001). IPFD accounted for 13.5% and 4.6% of the variance in total pancreatic T1 in the most adjusted and unadjusted models, respectively (Figure 4). The median splenic T1 was 1532.25 ms (IQR: 1434.88 to 1631.75 ms), with no significant association found between splenic T1 and IPFD.

### 3.3. Associations of Intrapancreatic Fat Deposition with Regional T1 of the Pancreas

The median T1 relaxation times were 1001.01 ms (IQR: 920.67 to 1103.36 ms) for the pancreatic head, 997.40 ms (IQR: 885.17 to 1084.12 ms) for the pancreatic body, and 992.58 ms (IQR: 920.30 to 1103.79 ms) for the pancreatic tail. In the most adjusted model, IPFD was significantly negatively associated with T1 values in the pancreatic head (β = –3.26, *p* = 0.03), body (β = –4.02, *p* = 0.004), and tail (β = –3.99, *p* = 0.002) (Table 2).

## 4. Discussion

T1 is a quantitative MRI parameter that has been investigated in relation to major pancreatic diseases, including chronic pancreatitis, pancreatic cancer, and T2DM. However, previous studies may have misestimated T1 values by failing to account for the influence of IPFD—a key contributor to pancreatic disease [7,18]. This study identified a significant link between IPFD and pancreatic T1, highlighting the impact of fat on pancreatic tissue properties. This association remained consistent across all pancreatic regions (head, body, and tail) and persisted after adjustment for covariates. By contrast, no significant association was observed between IPFD and splenic T1 across both the unadjusted and all adjusted models. Splenic T1 is less susceptible to fat-related signal confounding than pancreatic T1 and, therefore, served as a pragmatic internal comparator to help determine whether the observed associations are specific to the pancreas. Overall, the present study strengthened prior evidence by (i) analysing head/body/tail and total pancreatic T1, (ii) using multivariable linear regression with adjustment for demographic, anthropometric, and metabolic covariates, and (iii) quantifying the incremental contribution of IPFD to pancreatic T1 variance.

Our main finding suggests that IPFD significantly affects T1 of the pancreas. This is consistent with the findings of an earlier study by Higashi et al. [14]. The authors evaluated the influence of IPFD (determined with the use of a Dixon sequence) on T1 of the pancreas in 45 participants and found a significant negative correlation between IPFD and T1 of the pancreas on non-fat-suppressed T1 mapping images [14]. Moreover, the correlation was still observed when fat within the pancreas was considered as a binary variable: T1 of the pancreas was significantly shorter in individuals with an IPFD of ≥ 10% than those with an IPFD of < 10% [14]. The present study used a complementary approach to measuring IPFD, the HISTO sequence, and enjoyed a larger sample size. The main finding may go some way to explain the inconsistent results of T1 of the pancreas studies in the past—even within the same disease setting (such as T2DM) [19]. Given that excessive IPFD is a key driver of pancreatic diseases (according to the PANDORA hypothesis), a shorter T1 of the pancreas would be expected in T2DM individuals. This was indeed shown in a study demonstrating that native T1 values of the pancreas were significantly lower in individuals with T2DM than in healthy individuals [20]. However, opposite findings were reported in other studies [7,18,21]. Yasokawa et al. and Saad et al. found that prediabetes and T2DM are associated with prolonged T1 of the pancreas in adults and children [7,18], whereas Ashihara et al. found that there was no significant association between T1 of the pancreas and prediabetes and T2DM [21]. The lattermost studies need to be interpreted with caution as the effect of IPFD on T1 of the pancreas was not accounted for.

An important practical implication of our findings is that pancreatic MRI protocols may benefit from further optimisation in future studies. Since fat in the body produces a high signal and appears bright in T1-weighted images, it could obscure the evaluation of underlying pathology such as inflammation and oedema [22]. This emphasises the need to use fat-saturated MRI protocols in clinical settings. Several approaches could be used to minimise the effect of excessive IPFD. The chemical shift selective (CHESS) imaging technique omits the signal from fat by removing the magnetisation of fat tissues before the signals from other tissues are acquired [23,24]. A more advanced approach employing spatial spectral pulses has recently been developed that selectively excites only water in the tissues [25]. However, the increased scan time of this pulse and its sensitivity to B0 inhomogeneities require a longer TR and wider spectral bandwidth, limiting its use in lower field strength [22,25]. Unlike CHESS-based techniques, the short-tau inversion recovery (STIR) imaging technique is insensitive to B0 inhomogeneity and allows fat saturation in the target organ [22,26]. However, the 180º inversion pulses used in STIR are not exclusively selective for fat and can inadvertently suppress other tissues with short T1 [23]. An alternative approach to achieve fat saturation is the Dixon method [22,23]. This technique exploits the chemical shift between fat and water and requires postprocessing to separate their respective signals [22,23]. Unfortunately, as a chemical-shift-based technique, the Dixon method is also sensitive to B0 inhomogeneities. Additionally, the prolonged scanning time may lead to degradation in acquired images [23]. However, advancements in phase correction techniques during data acquisition and postprocessing have significantly minimised these adverse effects. One modification is the ‘three-point’ Dixon method developed by Glover and Schneider, which addresses B0 inhomogeneity issues through a phase unwrapping algorithm [27].

A strength of the present study is the use of a 3.0 T scanner—providing a more accurate evaluation compared to a 1.5 T scanner [28]. A low signal-to-noise ratio (SNR) particularly affects the low-fat content areas as a result of the skewed noise distribution from the calculation of signal intensity [29]. It may therefore lead to a misestimation of the low-fat content of the pancreas. Using a 3.0 T scanner provides a higher SNR than 1.5 T [28,30]. Moreover, the present study adjusted for body composition, demographic characteristics, as well as glucose and lipid metabolism biomarkers (i.e., parameters known to affect IPFD)—something that was not done in previous studies [2,13]. Several limitations of the present study also need to be acknowledged. First, although the HISTO sequence can provide a more direct measurement of IPFD, only one voxel was used in the present study. However, a previous study showed that there were no significant differences in terms of IPFD between the regions of the pancreas [16]. We did not investigate other factors that may influence pancreatic T1 relaxation times, such as pancreatic iron content and protein composition [31]. Iron can affect T1 by contributing to oxidative stress, which may in turn increase IPFD [17]. Additionally, tissue iron content has been shown to impact T1 mapping [32]. Proteins can also influence T1, as their concentration affects the free-water-to-bound-water ratio and the total surface area of protein macromolecules [33,34]. Third, we did not compare T1 mapping with and without fat suppression. Future studies should explore these factors to better understand their effects on pancreatic T1 measurements. Last, as T1 was sampled from two selected slices using three 100 mm^2^ ROIs rather than whole-organ voxel-wise mapping, this approach may introduce sampling bias and may not fully capture variation in T1 within the pancreas. Therefore, regional estimates should be interpreted as representative sampled measures rather than an exhaustive characterisation.

In conclusion, this study demonstrated that both regional and total pancreatic T1 relaxation times are significantly and consistently influenced by IPFD. These findings highlight the need for a cautious interpretation of previous T1 studies in diseases of the pancreas. Further prospective studies involving populations with established pancreatic diseases are warranted.

## Figures and Tables

**Table 1:** Characteristics of study participants.

Characteristic	*n* = 124
Age (years)	57 (40–67)
Sex, *n* (%)	
Men	67 (54%)
Women	57 (46%)
Waist–height ratio	0.54 (0.47–0.61)
HbA1c (mmol/mol)	37.00 (34.00–41.00)
Glucose (mmol/L)	5.20 (4.80–5.65)
HDL cholesterol (mmol/L)	1.40 (1.10–1.80)
Triglyceride (mmol/L)	1.20 (0.80–1.70)

*Footnote*: Data are presented as median (interquartile range) or number (percentage). *Abbreviations*: HbA1c, glycated haemoglobin; HDL, high-density lipoprotein.

**Table 2:** Associations between intrapancreatic fat deposition and T1 of the pancreas and the spleen.

T1 Relaxation Time (ms)	Model	IPFD (%)			
		β	Lower CI	Upper CI	*p*
**Total pancreas**	1	−2.221	−4.036	−0.406	**0.017**
	2	−2.628	−4.558	−0.699	**0.008**
	3	−2.867	−4.938	−0.796	**0.007**
	4	−2.957	−5.080	−0.834	**0.007**
	5	−3.708	−5.876	−1.541	**0.001**
**Pancreas head**	1	−1.341	−3.792	1.110	0.281
	2	−2.153	−4.738	0.431	0.102
	3	−2.414	−5.197	0.369	0.088
	4	−2.394	−5.254	0.466	0.100
	5	−3.263	−6.234	−0.291	**0.032**
**Pancreas body**	1	−2.172	−4.412	0.067	0.057
	2	−2.662	−5.042	−0.282	**0.029**
	3	−2.966	−5.522	−0.411	**0.023**
	4	−3.211	−5.819	−0.603	**0.016**
	5	−4.019	−6.708	−1.330	**0.004**
**Pancreas tail**	1	−3.223	−5.243	−1.203	**0.002**
	2	−3.135	−5.292	−0.977	**0.005**
	3	−3.309	−5.613	−1.006	**0.005**
	4	−3.377	−5.716	−1.038	**0.005**
	5	−3.985	−6.413	−1.556	**0.002**
**Spleen**	1	0.418	−2.258	3.094	0.757
	2	0.520	−2.322	3.361	0.718
	3	0.941	−2.110	3.993	0.542
	4	1.184	−1.927	4.294	0.452
	5	1.208	−2.107	4.524	0.472

*Footnotes*: Data are presented as β coefficients, 95% confidence intervals, and *p* values from linear regression models. Statistically significant values (*p* < 0.05) are shown in bold. Model 1, unadjusted. Model 2, adjusted for age and sex. Model 3, adjusted for age, sex, ethnicity, and waist–height ratio. Model 4, adjusted for age, sex, ethnicity, waist–height ratio, glucose, and glycated haemoglobin. Model 5, adjusted for age, sex, ethnicity, waist–height ratio, glucose, glycated haemoglobin, high-density lipoprotein cholesterol, and triglyceride. Data for glucose, glycated haemoglobin, high-density lipoprotein cholesterol, and triglyceride were log-transformed. *Abbreviations:* CI, confidence interval; IPFD, intrapancreatic fat deposition.

## Data Availability

The original contributions presented in this study are included in the article. Further inquiries can be directed to the corresponding author.

## Abbreviations

The following abbreviations are used in this manuscript:

CHESS	Chemical shift selective
CI	Confidence interval
HbA1c	Glycated haemoglobin
HDL	High-density lipoprotein
HISTO	High-speed T2-corrected multi-echo
IPFD	Intrapancreatic fat deposition
IQR	Interquartile range
MRI	Magnetic resonance imaging
PANDORA	PANcreatic Diseases Originating from intrapancReatic fAt
ROI	Region of interest
SNR	Signal-to-noise ratio
STIR	Short-tau inversion recovery
T1	Tissue longitudinal relaxation time
T2DM	Type 2 diabetes mellitus
VIBE	Volumetric interpolated breath-hold examination
